# Methane mitigation, nutritional evaluation, and in vitro degradation kinetics of diets based on tropical grass haylage treated with xylanase

**DOI:** 10.1007/s11250-026-05183-7

**Published:** 2026-06-22

**Authors:** Ana Beatriz de Almeida Duarte, Tairon Pannunzio Dias-Silva, Wesleyson Cristian Correa Viana, Antonio Leandro Chaves Gurgel, Marcos Jácome de Araújo, Adibe Luiz Abdalla, Leílson Rocha Bezerra, Ricardo Loiola Edvan, Romilda Rodrigues Nascimento, Sheila Vilarindo de Sousa, Carlo Aldrovandi Torreão Marques, Luis Carlos Vinhas Ítavo, Paulo de Mello Tavares de Lima

**Affiliations:** 1 Campus Professora Cinobelina Elvas, Universidade Federal do Piauí, Bom Jesus, PI Brasil; 2 Centro de Energia Nuclear na Agricultura, Universidade de São Paulo, Piracicaba, SP Brasil; 3 Centro de Saúde e Tecnologia Rural, Universidade Federal de Campina Grande, Campina Grande, PB Brasil; 4 Escola Agrícola de Jundiaí, Universidade Federal do Rio Grande do Norte, Natal, RN Brasil; 5 Faculdade de Medicina Veterinária e Zootecnia, Universidade Federal de Mato Grosso do Sul, Campo Grande, MS Brasil; 6 College of Agriculture, Life Sciences and Natural Resources, Department of Animal Science, University of Wyoming, Laramie, WY USA

**Keywords:** Fermentation, Fiber degradability, Gas production, Methane yield, Ruminants

## Abstract

The aim of this study was to evaluate the effect of xylanase addition (1.8 g/kg dry matter [DM]) to tropical grass haylage used in total mixed rations (TMR) on in vitro organic matter degradability, ruminal fermentation kinetics, and methane (CH4) emission. A completely randomized design was adopted in a 4 × 2 factorial arrangement, corresponding to four tropical grass cultivars (Andropogon, Mombaça, Massai, and Marandu) with or without xylanase addition and five replications per treatment (haylage bales), totaling 40 experimental bales. Ninety days after baling, the bales were opened and used to formulate total mixed rations, which were used as substrates for the in vitro incubations, according to the experimental treatments, totaling 40 TMR units (experimental diets). Chemical composition and ruminal fermentation parameters differed among grass cultivars. TMR based on Andropogon haylage showed greater crude protein concentration and higher acetate production (*P* < 0.0001), whereas Mombaça haylage promoted greater propionate production and a more favorable acetate: propionate ratio (*P* < 0.0003). The addition of xylanase did not alter organic matter degradability or total gas production (*P* > 0.05). However, xylanase tended to reduce CH4 emission (*P* = 0.0882) and improve CH4 efficiency (*P* = 0.0701), indicating a potential mitigation effect on enteric methane production. Diets based on Massai haylage without xylanase showed lower gas production and faster fractional fermentation rates. Overall, the results indicate that tropical grass haylage can be successfully used in TMR systems, and xylanase supplementation may contribute to improved ruminal fermentation efficiency and methane mitigation, although further studies evaluating different enzyme levels are still required.

## Introduction

Climatic seasonality in tropical regions causes marked variations in forage quantity and quality, increasing fiber concentration and reducing feed digestibility (Ku-Vera et al. 2020). These changes negatively affect ruminant performance and production efficiency (Cheng et al. 2022).

In this climatic scenario, during the dry season, forage availability and nutrient supply decline substantially because tropical grasses accumulate large amounts of poorly digestible fiber carbohydrates (Meale et al. 2014; Franco et al. 2021). The plant cell wall represents a major challenge for ruminal degradation because it is composed of complex structural carbohydrates, including cellulose, hemicellulose, and lignin, whose proportions vary according to plant species and maturity stage (Jørgensen et al. 2007).

In diets with a high fiber content, enteric CH_4_ production tends to increase (Ruviaro et al. 2015). Microorganisms such as methanogenic archaea (chemotrophic rumen bacteria) use carbon dioxide (CO_2_) and hydrogen (H_2_), produced after the fermentation process, to form CH_4_ (Patra et al. 2017). According to Beauchemin et al. (2020), in diets based on low-quality forage, the CH4 released represents an energy loss for the animal (3–17%), reducing feed efficiency. In addition, CH_4_ is a greenhouse gas (GHG), with the ability to retain energy 25 times greater than CO_2_ (IPCC 2007), and is responsible for approximately 5% of global anthropogenic GHG emissions (Shukla et al. 2022).

Ensuring the availability of high-quality forage during the dry season requires efficient forage conservation strategies (Balehegn et al. 2022). In this context, haylage has emerged as an alternative conservation method for tropical grasses because it reduces field losses and preserves forage quality. Haylage is produced by partially dehydrating the forage to dry matter contents between 400 and 600 g/kg before storage (Edvan et al. 2023; Duarte et al. 2024).

Several factors influence enteric CH_4_ production (Abdalla et al. 2012; Cerri et al. 2016). Low-digestibility diets lead to increased gas emissions from enteric fermentation; animals slaughtered at older ages spend more time on pasture, consequently contributing to higher GHG emissions; and less efficient production systems characterized by low slaughter weights result in increased GHG emissions per kg of meat or milk produced (Gerber et al. 2013a). Thus, the rumen fermentation process (exogenous fibrolytic enzymes, ionophores, halogenated compounds, secondary plant compounds, lipids, nitrooxy compounds, propionate precursors, chemical and physical properties of diets) can be manipulated to reduce enteric CH_4_ emission. (Beauchemin et al. 2008; Cottle et al. 2011; Gerber et al. 2013b; Wang et al. 2023).

Many in vitro studies have shown the efficiency of exogenous fibrolytic enzymes in degrading the fiber fraction of different substrates with consequent improvement in nutritional quality (Soltan et al. 2013; Tang et al. 2013; Adesogan et al. 2014; Meale et al. 2014; Romero et al. 2015; Díaz et al. 2015; Elghandour et al. 2016; Gado et al. 2017; Kholif et al. 2017; Hernández et al. 2017; Vallejo-Hernández et al. 2018; Sakita et al. 2020). Based on these findings, it was hypothesized that xylanase enzyme, added in the pre-drying process of tropical grasses, would support the degradation process of organic matter, making greater quantities of nutrients available. This would result in direct benefits such as the reduction of enteric CH_4_ emission by ruminants and greater energy use from the diet.

Given this scenario and the increasing interest in haylage as a method for preserving forage during periods of scarcity, this study aimed to evaluate the effect of adding xylanase to tropical grass haylages used in total mixed rations (TMR) for ruminants, on in vitro degradability of organic matter, rumen degradation kinetics, and CH_4_ emission.

## Materials and methods

The experimental procedures of the present research involving animals were approved by the institutional committee for the use of animals in research at the Center for Nuclear Energy in Agriculture, at the University of São Paulo (Protocol 011/2016).

### Experiment location

The experiment was initially conducted in Bom Jesus, Piauí, Brazil, with subsequent evaluations at the Animal Nutrition Laboratory of the Center for Nuclear Energy in Agriculture at the University of São Paulo. The city of Bom Jesus is located in the south of the state of Piauí, in the Alto-Médio Gurguéia microregion, at latitude 09º04’28” south and longitude 44º21’31” west, at an altitude of 277 m. The study region is classified asAw, according to the Köppen classification, described by Medeiros et al. (2013) and Alvares et al. (2013), with summer rains and a dry period of approximately six months. The grasses evaluated and used in the experiment came from a pasture area established in 2016, located in the Agrostological Field of the Professor Cinobelina Elvas Campus (CPCE) of the Federal University of Piauí (UFPI).

### Grasses, experimental design, haylage production and experimental diets (substrates)

Four tropical grasses (*Andropogon gayanus* cv. Planaltina; *Panicum maximum* cv. Mombaça; *Panicum maximum* cv. Massai, and *Urochloa brizantha* cv. Marandu) were used for haylage production. A completely randomized design was used in a 4 × 2 factorial scheme, corresponding to four cultivars and enzyme addition (with or without xylanase), and five replications per treatment (haylage bales), totaling 40 experimental bales with approximate dimensions of 20 cm × 25 cm × 16 cm (width, length, and height).

The pastures were irrigated and mowed uniformly at the beginning of the experiment following the management recommendation for each species, and fertilization was done by soil analysis and recommendations for demanding species (Martha Jr et al. 2007). Forage species were cut at the phenological stage before flowering to determine the chemical composition (Table 1) and haylage production. The harvested material was placed on a plastic canvas and sun dried until it reached 50% moisture content (Edvan et al. 2023; Duarte et al. 2024). During the sun exposure time, the forage mass was turned every 20 min to standardize dehydration. When the material reached the appropriate level of moisture, it was collected for baling in manual balers.

**Table 1:** Chemical composition (g/kg of dry matter, DM) of the fresh ingredients that made up the total mixed ration (TMR)

g/kg DM	Andropogon	Mombaça	Massai	Marandu	Ground corn	Soybean meal
Dry matter*	365.3	316.0	334.8	284.7	885.1	896.2
Crude protein	79.3	63.0	76.1	69.8	121.6	418.5
Ash	42.0	63.8	55.4	57.5	20.7	67.5
Neutral detergent fiber	758.4	779.5	794.8	703.3	169.5	147.8
Acid detergent fiber	363.6	419.7	422.6	352.5	54.1	87.2

*g/kg fresh matter; DM – Dry matter

Before baling, the xylanase was added in the test treatments at a level of 1.8 g/kg dry matter (DM), as recommended by the manufacturer. Thus, each grass cultivar was subjected to two treatments: with or without xylanase addition (Fibrozyme^®^ - ALLTECH). The product was extracted from the fungi *Aspergillus niger* and *Trichoderma longibrachiatum*. The product was composed of xylanase and a surfactant (*Yucca echtigera* extract), a vehicle to increase the contact between the enzymes and the substrate. After pre-drying (500 g Kg^-1^ DM) (Edvan et al. 2023; Duarte et al. 2024) followed by the microwave oven method (Souza et al. 2002) the forage was baled in homemade rectangular balers at an approximate weight of 2 kg per bale.

The bales were opened 90 days after baling. A total mixed ration (TMR) was formulated with a forage: concentrate ratio of 60:40 to be used as a substrate in the in vitro incubation. The concentrate was made of ground corn and soybean meal, following a 70:30 ratio (DM basis).

### Chemical analysis

All samples of the different grass haylages were dried in an air circulation and renewal oven (MARCA), at 55 °C for 72 h and ground in a Wiley-type mill with a 1-millimeter sieve (Marconi, Piracicaba, SP, Brazil). Dry matter (method no. 934.01), ash (method no. 942.05), crude protein (CP; method no. 2001.11), and ether extract (EE; method no. 920.39) contents were determined according to the Association of Official Analytical Chemists (AOAC 2011). Ash was used to calculate the organic matter (OM) content. Neutral detergent fiber (NDF), acid detergent fiber (ADF), hemicellulose and cellulose were determined according to the methodologies described by Van Soest et al. (1991) and adapted by Mertens (2003). To estimate total carbohydrates (TCHO), the equation proposed by Sniffen et al. (1992) was used: TCHO = 100 - (%CP + % EE + %Ash).

### In vitro gas production

The in vitro evaluation of gas production was carried out at the Animal Nutrition Laboratory of the Center for Nuclear Energy in Agriculture, in Piracicaba, São Paulo, Brazil. In vitro gas production and degradability were determined following the procedures of Theodorou et al. (1994), using a semi-automatic system, as described by Mauricio et al. (1999) with adaptations previously described by Abdalla et al. (2012) and Bueno et al. (2005).

Three rumen-cannulated male Santa Inês sheep (60 ± 2.5 kg body weight) were used as rumen fluid donors. The animals had *ad libitum* access to mineral supplements, water, and Tifton-85 hay (*Cynodon spp*.) with a concentrated mixture (70% ground corn and 30% soybean meal). The rumen contents of each animal were collected before morning feeding and maintained at 39 °C under anaerobic conditions until inoculation. Two inoculum per substrate were prepared, using a solid: liquid ratio of 50:50 (Bueno et al. 2005). For each substrate, two bottles were incubated (location 1 and location 2) (experimental unit), and each experimental unit was incubated in each one of the three inocula. Gas pressure (psi) was measured using a pressure transducer and a datalogger (Mauricio et al. 1999) at 2, 4, 8, 12, and 24 h after closing the bottles to estimate the total gas produced (mL) using the equation obtained with linear regression (*n* = 500; R^2^ = 0.99), as described by Soltan et al. (2013).

After each gas pressure measurement, a gas subsample (2.5 mL) was collected into 10-mL vacuum tubes to determine CH_4_ concentration using a gas chromatograph (Shimadzu GC 2014, Chiyodaku, Tokyo, Japan) equipped with a flame ionization detector (FID) and an HP-mole sieve capillary column (GC 30 m × 0.53 mm × 25 μm). GC conditions were a column temperature of 60 °C, injector temperature of 200 °C, detector temperature of 240 °C, and carrier gas (helium) in constant flow at 10 mL/min. After each gas pressure measurement and collections, bottles were vented with a needle to release excess pressure and to allow a new accumulation of gas in the subsequent time, and consequently measure the microbial activity in that incubated substrate.

The CH_4_ emission estimate was calculated according to Longo et al. (2006): CH_4_ (mL/g of DM) = (VGP + US)*CH_4(%)_, where VGP is the volume of gas produced (mL); US is the upper space of the bottle (85 mL); CH_4(%)_ is the percentage of CH_4_ in the subsample; and CH_4_ is the concentration of CH_4_ (mL/g DM). Methane efficiency (%) was calculated as (CH_4_/VGP) and expresses the proportion of CH_4_ in each mL of gas produced.

### Organic matter degradability parameters and rumen fermentation

After 24 h, the bags were removed from the bottles and both were immediately immersed in cold water (-4 °C) to inhibit further fermentation. The period of 24 h was selected based on previous research, which demonstrated that a plateau in results can be achieved within this period if the substrate particle size is approximately 1 mm (Yáñez-Ruiz et al. 2016). All bags were treated with a neutral detergent solution for one hour at 90 °C and then washed with hot water and acetone in sequence. The bags were placed in a muffle furnace at 550 °C for 4 h, and the ashes were weighed and used to determine the truly degraded organic matter (TDOM), being expressed as a degradability coefficient according to Wiseman (2018).

Partition factor (PF) was calculated using the ratio between TDOM (in mg) and total gas production (in mL) over a 24-hour incubation period (Blümmel et al. 1997). The PF reflects the proportion of TDOM that can be used by rumen microorganisms in relation to the amount of gas produced during the incubation period. After in vitro fermentation, a 2-mL subsample of the fermentation fluid was collected from each bottle to determine the concentration of short-chain fatty acids (SCFA), using the methodology of Palmquist and Conrad (1971), with gas chromatography (CG 2014 Shimadzu, Tokyo, Japan) equipped with FID and the PG 10% SP-1200/1 H3PO4 80/100 Chromosorc WAW column (ct. no. 11965, stainless steel 60 × 1/800, SUpelco, Bellefonte, PA, USA) as described by Lima et al. (2018).

The potential cumulative gas produced (A) (mL), lag time (L; the initial time before degradation start), T_1/2_ (time (h) to obtain the half value of A), and the fractional rate of fermentation (µ_1/2_) were calculated using the generalized Mitscherlich model as proposed by France et al. (1993), using the NLIN procedure (v. 9.4, SAS Inc., Cary, NC, USA). The maximum number of iterations used was 100.

$$Y = A\{ 1-exp[ - b(t-L)-c(\surd t - \surd L)]\}$$

where Y is the cumulative gas production (in mL), b is the fractional rate of gas production, t is the incubation time (in hours), L is the lag time (in hours), and c is the shape parameter.

### Statistical analysis

Data were initially evaluated for normality of residuals using the Shapiro–Wilk test and for homogeneity of variances using Bartlett’s test. Residual plots were also visually inspected to confirm model assumptions. When necessary, data transformations were considered; however, all variables met the assumptions required for analysis of variance, and therefore the original data were used. Data were analyzed using the GLM procedure of SAS (v. 9.4, SAS Institute Inc., Cary, NC, USA) in a completely randomized design with a 4 × 2 factorial arrangement. The first factor corresponded to four tropical grass haylages: *Andropogon gayanus* cv. Planaltina, *Panicum maximum* cv. Mombaça, *Panicum maximum* cv. Massai and *Urochloa brizantha* cv. Marandu, the second factor was the addition or not of xylanase. When significant by the F test (*P* < 0.05), the effects of the sources of variation and their interactions were analyzed by the Tukey’s test. When the interaction was statistically significant, the unfolding was performed and described in the results. Significance was declared at *P* < 0.05 and trends at 0.05 ≤ *P* < 0.10.

## Results

The DM content of the TMR was significantly affected (*P <* 0.0001) by the cultivar × enzyme interaction, with higher DM for the TMR based on Mombaça grass haylage with xylanase (Table 2). The data show that the CP content differed (*P <* 0.0001) among diets, with highest and lowest values for the TMR based on Andropogon and Massai grass haylage, respectively.

**Table 2:** Chemical composition (g/kg of dry matter) of the total mixed ration (TMR) based on tropical grass haylage with the addition of xylanase

Treatments	Total mixed ration					*P*-Value		
	Andropogon	Mombasa	Massai	Marandu	mean	TMR	Enzyme	Interaction
	**Dry matter (g/kg fresh matter)**							
With enzyme	651.8Ab	750.9Aa	664.9Ab	629.5Ac		< 0.0001	< 0.0001	< 0.0001
Without enzyme	660.1Aa	599.9Bc	640.5Bb	598.5Bc				
	**Crude protein**							
With enzyme	146.6	128.1	121.9	131.9	132.1	< 0.0001	0.7217	0.0526
Without enzyme	134.7	131.2	125.0	134.2	131.3			
Mean	140.6a	129.7bc	123.4c	133.0ab				
	**Ether extract**							
With enzyme	4.06Ab	2.91Ad	5.35Aa	3.59Ac		< 0.001	< 0.001	< 0.001
Without enzyme	2.92Bb	2.94Ab	4.01Bab	2.99Bb				
	**Neutral detergent fiber**							
With enzyme	689.6Aa	477.1Ab	498.3Ab	531.0Ab		0.0006	0.0462	0.0023
Without enzyme	563.9Ba	534.1Aa	573.5Aa	434.2Aa				
	**Acid detergent fiber**							
With enzyme	257.4	282.9	237.3	226.0	250.9	0.0103	0.1403	0.9745
Without enzyme	236.7	268.4	227.7	213.3	236.5			
Mean	247.0ab	275.6a	232.5b	219.6b				
	**Ash**							
With enzyme	52.35	71.80	57.90	56.85	59.6	< 0.0001	0.1199	0.6107
Without enzyme	52.20	59.30	5.480	54.80	57.3			
Mean	52.21b	65.57a	56.34b	55.83b				
	**Total Carbohydrates**							
With enzyme	620.4Aa	508.4Ab	612.8Aa	633.1Aa		0.0246	0.0087	0.0017
Without enzyme	659.0Aa	651.8Ba	608.5Aa	600.4Aa				

Means followed by different lowercase letters in the rows and uppercase letters in the columns differ significantly according to the Tukey test at 5% probability; The absence of letters following the means of the rows and columns indicates the non-occurrence of a significant effect of TMR, Enzyme or their interaction according to the Tukey test at 5% probability; Interaction - interaction between total mixed ration and enzyme

TMR × enzyme interaction also significantly affected ether extract (*P <* 0.0001) and NDF (*P* = 0.0023) contents. The TMR based on Andropogon grass haylage with xylanase showed higher fiber content compared to other diets, and the highest content of ether extract was found in the TMR based on Massai haylage with and without xylanase. The ADF fraction was affected by TMR, with a higher proportion in diets with Andropogon and Mombaça grass haylage. The proportion of ash was higher in the TMR based on Mombaça grass haylage with xylanase. TCHO differed depending on the TMR x enzyme interaction, with a lower proportion in the diet with Mombaça grass haylage without enzyme.

No interaction effect (*P* > 0.05) between TMR and enzyme supplementation was observed for gas production parameters, CH4 emission, or in vitro OM degradability (Table 3). There was also no effect of TMR alone on these variables (*P >* 0.05). However, the xylanase tended to reduce CH_4_ emission (*P =* 0.0882) and increase CH_4_ efficiency (*P =* 0.0701) in TMR based on tropical grass haylage.

**Table 3:** In vitro degradability of organic matter, total gas production and methane emission in total mixed ration (TMR) based on tropical grass haylage with the addition of xylanase

Treatments	Total mixed ration					*P*-Value		
	Andropogon	Mombaça	Massai	Marandu	Mean	TMR	Enzyme	Interaction
	**In vitro degradability of organic matter (g Kg** ^− 1^ **)**							
With enzyme	460.8	498.4	487.7	511.1	489.5	0.6477	0.7090	0.7131
Without enzyme	483.6	465.3	481.7	499.7				
Mean	472.2	481.8	484.7	505.4	482.6			
	**In vitro gas production (mL g OMD** ^**− 1**^ **)**							
With enzyme	186.1	204.2	186.4	175.3	188.0	0.2475	0.1678	0.9684
Without enzyme	198.9	224.4	193.4	195.2	203.0			
Mean	192.5	214.3	189.9	185.2				
	**Methane emission (mL g OMD** ^**− 1**^ **)**							
With enzyme	74.22	72.16	44.25	52.67	60.82B	0.5564	0.0882	0.8770
Without enzyme	82.14	87.21	73.91	81.77	81.26 A			
Mean	78.18	79.68	59.08	67.22				
	**Methane efficiency (%)**							
With enzyme	37.31	33.32	22.84	28.186	30.41B	0.4950	0.0701	0.8055
Without enzyme	40.59	38.32	35.89	41.15	38.99 A			
Mean	38.95	35.82	34.66	29.36				
	**Partitioning factor**							
With enzyme	5.65	5.13	5.51	5.94	5.56	0.4788	0.1133	0.9691
Without enzyme	5.10	4.55	5.24	5.18	5.02			
Mean	5.37	4.84	5.38	5.56				

P value < 0.05 indicates significant difference; *p* value > 0.05 and < 0.1 indicates a tendency to differ significantly; the absence of letters following the means in the rows and columns indicates the non-occurrence of a significant effect of TMR, Enzyme or their interaction according to the Tukey test; It was used lowercase letters in the rows and uppercase letters in the columns; OMD = organic matter degraded; Interaction - interaction between total mixed ration and enzyme

The total concentration of SCFA was influenced (*P <* 0.015) by the grass haylage used in the TMR, but not by the inclusion of the enzyme or their interaction, with a greater quantity of these observed in the TMR with Andropogon grass haylage compared to the TMR based on Mombaça grass haylage (Table 4). The proportion of acetate was highest (*P <* 0.0001) in the TMR with Andropogon grass haylage. On the other hand, the highest proportions of propionate (*P <* 0.0003) and butyrate (*P <* 0.0001) were observed in the diet with Mombaça grass haylage compared to the diet containing Andropogon grass haylage, resulting in the different (*P <* 0.0001) acetate: propionate ratio between these substrates.

**Table 4:** Concentration of short-chain fatty acids (mmol L^− 1^) in the total mixed ration (TMR) based on tropical grass haylage with the addition of xylanase

Treatments	Total mixed ration					*P*-Value		
	Andropogon	Mombaça	Massai	Marandu	Mean	TMR	Enzyme	Interaction
	**Total short-chain fatty acids**							
With enzyme	142.4	126.0	131.2	139.4	134.8	0.0152	0.1770	0.8942
Without enzyme	154.3	132.9	133.1	144.1	141.1			
Mean	148.3a	129.4b	132.2ab	141.7ab				
	**Acetic acid**							
With enzyme	58.69a	51.94b	53.02ab	54.39ab	54.52	< 0.0001	0.7075	0.6733
Without enzyme	58.00a	51.10b	54.20ab	56.14ab	54.86			
Mean	58.34a	51.55c	53.64bc	55.25ab				
	**Propionic acid**							
With enzyme	23.14bc	28.20ab	27.21abc	26.94abc	26.37	0.0003	0.6366	0.8803
Without enzyme	22.72c	28.95a	26.19abc	25.90abc	25.94			
Mean	22.91b	28.53a	26.73a	26.44a				
	**Butyric acid**							
With enzyme	13.02bc	14.17ab	14.10ab	13.23bc	13.63	< 0.0001	0.7895	0.3734
Without enzyme	13.30abc	14.44a	13.88abc	12.69c	13.58			
Mean	13.14b	14.35a	13.91a	12.94b				
	**Isobutyric acid**							
With enzyme	0.788	1.064	1.000	0.916	0.942	0.8870	0.5559	0.6670
Without enzyme	1.061	1.002	0.975	0.983	1.000			
Mean	0.924	1.031	0.987	0.949				
	**Isovaleric acid**							
With enzyme	3.019	3.223	3.248	3.139	3.157	0.7020	0.6392	0.3981
Without enzyme	3.474	3.131	3.323	2.973	3.225			
Mean	3.246	3.177	3.286	3.056				
	**Valeric acid**							
With enzyme	1.332	1.402	1.408	1.369	1.377	0.2453	0.8938	0.1585
Without enzyme	1.433	1.364	1.435	1.295	1.381			
Mean	1.382	1.383	1.421	1.332				
	**Acetate/propionate ratio**							
With enzyme	2.700a	1.848b	1.967ab	2.063ab	2.144	< 0.0001	0.5613	0.8858
Without enzyme	2.753a	1.771b	2.100ab	2.262ab	2.221			
Mean	2.726a	1.809b	2.033b	2.162b				

Means followed by different lowercase letters in the rows and uppercase letters in the columns differ significantly according to the Tukey test at 5% probability; The absence of letters following the means in the rows and columns indicates the non-occurrence of a significant effect of TMR, Enzyme or their interaction according to the Tukey test at 5% probability; Interaction - interaction between total mixed ration and enzyme

A significant effect of interaction between TMR and enzyme was found on potential gas produced (*P =* 0.0377) and fractional rate of fermentation (*P =* 0.0104) (Table 5). In this case, after unfolding the interaction, it was found that the TMR based on Massai grass haylage without xylanase produced the lowest volume of gas and the fastest fractional fermentation rate compared to other diets. Lag time and T_1/2_ did not differ (*P >* 0.05) among evaluated diets.

**Table 5:** Kinetics of rumen degradation of total mixed ration (TMR) based on tropical grass haylage with the addition of xylanase

Treatments	Total mixed ration					*P*-Value		
	Andropogon	Mombaça	Massai	Marandu	Mean	TMR	Enzyme	Interaction
	**A (potential gas produced**,** mL)**							
With enzyme	370.1Aa	243.0Aa	317.7Aa	269.8Aa		0.2014	0.9457	0.0377
Without enzyme	309.0Aab	469.5Aa	150.5Bb	283.1Aab				
	**L (lag time**,** h)**							
With enzyme	0.786	0.778	0.856	0.709	0.782	0.4021	0.1613	0.7990
Without enzyme	0.659	0.772	0.737	0.666	0.709			
Mean	0.723	0.775	0.797	0.688				
	**T**_**1/2**_ ** (the time (h) at half of A)**							
With enzyme	10.44	7.63	9.39	9.02	9.12	0.3809	0.3793	0.1106
Without enzyme	7.86	12.56	7.75	7.34	7.88			
Mean	9.15	10.10	6.57	8.18				
	**µ**_**1/2**_** (the fractional rate of fermentation**,** mL h**^**− 1**^**)**							
With enzyme	0.012Aa	0.019Aa	0.015Ba	0.014Aa		0.0263	0.1093	0.0104
Without enzyme	0.018Aab	0.007Ab	0.039Aa	0.016Ab				

Means followed by different lowercase letters in the rows and uppercase letters in the columns differ significantly according to the Tukey test at 5% probability;

The absence of letters following the means in the rows and columns indicates the non-occurrence of a significant effect of TMR, Enzyme or theeir interaction according to the Tukey test at 5% probability; Interaction - interaction between total mixed ration and enzyme

## Discussion

### Chemical composition

There is a consensus in the literature that nutrient availability from forages used to feed ruminants, the rate of nutrient degradation, total gas production, and CH_4_ emissions are influenced by both the type of substrate and the level of exogenous enzymes (Soltan et al. 2013; López et al. 2016; Vallejo et al. 2016; Kholif et al. 2017; Sakita et al. 2020). A higher CP content was found in the TMR based on Andropogon grass haylage. Edvan et al. (2024) found 92.1, 140.0 and 127.2 g of CP/kg DM in the Andropogon, Massai and Marandu grass haylages, respectively. In another study, Edvan et al. (2023) found 94.6 g of CP/kg DM in the Mombaça grass haylage. In the present study, one aspect to be considered was that the Andropogon grass presented the highest CP content among the grasses evaluated, both fresh and in TMR. It is important to consider that the quality of haylage is directly related to the practices of formation, establishment and maintenance of pasture areas, considering the recommendations for each species. Factors such as plant age, leaf/stem ratio, presence of nitrogen fertilization and environmental cultivation conditions interfere with the CP content of grasses. Among these, the leaf/stem ratio may probably have influenced, since the other factors were exactly similar among the cultivars evaluated.

The greater crude protein concentration observed in the Andropogon-based diets may have favored microbial activity and contributed to differences in ruminal fermentation among treatments. Adequate crude protein supply is essential to support rumen microbial growth and optimize fiber degradation (Van Soest 1994). In addition, the variation in the chemical composition of grasses is evident due to the different morphophysiological characteristics imposed by the different genera (Batistoti et al. 2012; Veras et al. 2020).

The haylage with xylanase associated with the concentrate, in the form of TMR, changed the fiber content of the different treatments, with the diet based on Andropogon grass haylage with xylanase having a higher fiber content. Diets based on Andropogon and Mombaça grasses had higher ADF contents, which may be related to the structural characteristics of these two forages, that have an erect growth habit, tall size, and large accumulation of stems (Euclides et al. 2015). The stem elongation rate is an important morphogenic characteristic of grasses with an upright growth habit (Rodrigues et al. 2008). This component increases yield and significantly interferes with the structure of the forage canopy, mainly in the leaf: stem ratio, which influences the material’s quality. According to Euclides (1995), NDF values below 55% are rare, values above 65% are common in new plant tissues and levels between 75 and 80% are found in plant tissues of advanced maturity. The NDF content is the most limiting factor in the consumption of roughage, with levels of cell wall constituents greater than 60% in DM correlating negatively with intake, making the NDF concentration the most consistently associated component with forage intake (Van Soest 1994). In addition, Massai had CP content below 70 g/kg DM, which can limit the activity of rumen microorganisms (Van Soest 1994; Sampaio et al. 2009).

### In vitro degradability, total gas production and methane emission

Xylanase supplementation did not improve the digestibility of the fiber fraction in the evaluated diets. This response may indicate that the enzyme level used was insufficient to substantially alter cell wall degradation under the conditions of the present study. It is believed that longer substrate incubation periods, the use of higher enzyme levels and the use of a blend of fibrolytic enzymes could promote a greater positive effect on the digestibility of the haylage. Therefore, more research should be conducted to fill these gaps. Antonio et al. (2018) used an enzyme blend and demonstrated more positive effects on the degradation of fiber fractions than the xylanase enzyme alone. Generally, the greater diversity of enzymes contained in the enzyme blend (for example, cellulase, hemicellulase, xylanases and glucanases) can explain its greater potential in the degradation of polysaccharides, increasing the digestibility of NDF, with a consequent greater availability of nutrients. Similar results were found by Feng et al. (1996) who reported an increase in the digestibility coefficient of DM and NDF, in a study evaluating the in vitro and in situ degradability with the addition of enzymes (50:50 of cellulase and xylanase), of dry, fresh and rehydrated forages, observing that the higher coefficients were detected for dry forages, which shows that the moisture content of the forage can influence the effectiveness of the enzymes.

Although organic matter degradability did not differ among treatments, diets supplemented with xylanase tended to reduce CH4 production and improve CH4 efficiency. This response may indicate a more efficient utilization of dietary substrates during ruminal fermentation. In general, diets with greater availability of digestible carbohydrates and lower concentrations of structural fiber tend to reduce methane production per unit of dry matter intake by favoring propionate production and reducing hydrogen availability for methanogenic archaea (Fouts et al. 2022). It is worth noticing that increased microbial fermentation provides greater production of final fermentation products, such as SCFA, hydrogen (H_2_) and carbon dioxide (CO_2_), which are substrates for the production of CH_4_, being responsible for losses on the order of 3–17% (Beauchemin et al. 2020), and for approximately 5% of global anthropogenic GHG emissions (Shukla et al. 2022).

The CH_4_ produced is the result of the inefficient conversion of energy potentially available in the TMR (McAllister and Newbold 2008). TMR with xylanase suggests better efficiency of substrate use, with lower CH_4_ production indicating consequent reduction in metabolic losses. However, results based on the use of this enzyme remain controversial. In an in vitro study, Elghandour et al. (2016) stated that the rumen fermentation is affected by both the rate and the time of application of the enzymatic additive. On the other hand, Sakita et al. (2020) conducted a trial evaluating the effect of five levels of fibrolytic enzyme extract (0, 5, 50, 500 and 5,000 µl), and found that the highest level of enzyme increased degradability, and total gas and CH_4_ production in the evaluated substrates.

These findings suggest that changes in fermentation patterns and nutrient degradability depend on both substrate characteristics and enzyme supplementation level (Soltan et al. 2013; López et al. 2016; Vallejo et al. 2016; Kholif et al. 2017; Sakita et al. 2020). The response to fibrolytic enzymes appears to depend on forage type, enzyme composition, and supplementation level. Therefore, additional studies evaluating different xylanase concentrations and enzyme combinations are warranted to better understand their effects on degradability, ruminal fermentation, and methane mitigation in tropical grass haylage diets. Furthermore, the high CP and low NDF diet formulation tended to produce less CH_4_ (Sakita et al. 2020), showing that CH_4_ production is influenced by forage quality (Soltan et al. 2013; Guyader et al. 2016; Gaviria-Uribe et al. 2020). Wang et al. (2023) reported that diets with high-quality forage generally promote an increase in the amount of gas produced. However, the amount of CH_4_ produced per unit of dry matter consumed decreases due to the higher rate of feed passage (Hammond et al. 2013). This implies that the provision of better-quality forage will increase the performance of the ruminant animal with a reduction in the production of CH_4_/unit of meat or milk, i.e. lower methane intensity (Beauchemin et al. 2020).

In addition to the use of fibrolytic enzymes, other management options can be adopted to reduce methane emissions and methane intensity in ruminant production, such as the use of ionophores, halogenated compounds, secondary plant compounds, lipids, nitrooxy compounds, propionate precursors, chemical and physical properties of diets (concentrates, forages, physical properties of diets and non-forage fiber sources) (Wang et al. 2023).

### Short-chain fatty acids and kinetics of rumen degradation

The influence of TMR on total SCFA production shows the direct effect of the substrate on the rumen fermentation pattern. Diets with a lower content of potentially digestible fiber combined with a fermentation of soluble carbohydrates resulting from the degradation of the cell wall (Romero et al. 2015), such as, TMR based on Mombaça haylage had greater content of propionate with direct effect and potential improvement in the acetate/propionate ratio in the rumen environment, indicating efficient use of the energy available in the rumen (Arriola et al. 2011) via microbial fermentation.

Due to the complex rumen environment, strategies to mitigate undesirable gases (CO_2_ and CH_4_) and enhance animal performance must be designed. Again, CH_4_ tended to be lower with the use of xylanase. However, more studies should be conducted, testing different levels of xylanase to determine the ideal level when using the product.

Diets based on Massai haylage without xylanase showed lower gas production potential and faster fractional fermentation rates than the other treatments. This shows that the fiber content implies a reduction in forage digestibility due to the presence of structural tissues (Leal et al. 2020; Molina-Botero et al. 2020; Valencia-Salazar et al. 2021), resulting in greater time for degradation of these components.

The similar lag times observed among diets suggest comparable patterns of microbial colonization and initial substrate degradation. The attachment of rumen microorganisms to feed particles represents the first step of the digestive process and can be influenced by particle structure and fiber composition (Hobson and Stewart 2012). Therefore, the absence of differences in lag time indicates that the evaluated diets provided similar conditions for the initial microbial adherence and colonization process.

## Conclusions

Xylanase supplementation at the evaluated level did not improve the degradability of diets based on tropical grass haylage. However, the enzyme showed potential to mitigate methane emission and improve fermentation efficiency. These findings reinforce the potential of tropical grass haylage as a sustainable feed resource for ruminant production systems. Further studies evaluating different xylanase levels and enzyme combinations are needed to optimize the response of tropical forage-based diets.

## Data Availability

The data that support the findings of this study are available upon request from the corresponding author. The data are not publicly available due to privacy or ethical restrictions.
